# Efficient Delivery of Dengue Virus Subunit Vaccines to the Skin by Microprojection Arrays

**DOI:** 10.3390/vaccines7040189

**Published:** 2019-11-20

**Authors:** David A. Muller, Alexandra C. I. Depelsenaire, Ashleigh E. Shannon, Daniel Watterson, Simon R. Corrie, Nick S. Owens, Christiana Agyei-Yeboah, Stacey T. M. Cheung, Jin Zhang, Germain J. P. Fernando, Mark A. F. Kendall, Paul R. Young

**Affiliations:** 1 Australian Infectious Diseases Research Centre, School of Chemistry and Molecular Biosciences, The University of Queensland, Brisbane, QLD 4072, Australia; 2 Australian Institute for Bioengineering and Nanotechnology, The University of Queensland, Brisbane, QLD 4072, Australia; depelsenaire@gmail.com (A.C.I.D.);; 3 Department of Chemical Engineering, ARC Centre of Excellence in Convergent BioNano Science and Technology, Monash University, Clayton, VIC 3800, Australia

**Keywords:** dengue virus, nanopatch, secreted E, virus challenge, microneedle, microarray patch, vaccine

## Abstract

Dengue virus is the most important arbovirus impacting global human health, with an estimated 390 million infections annually, and over half the world’s population at risk of infection. While significant efforts have been made to develop effective vaccines to mitigate this threat, the task has proven extremely challenging, with new approaches continually being sought. The majority of protective, neutralizing antibodies induced during infection are targeted by the envelope (E) protein, making it an ideal candidate for a subunit vaccine approach. Using truncated, recombinant, secreted E proteins (sE) of all 4 dengue virus serotypes, we have assessed their immunogenicity and protective efficacy in mice, with or without Quil-A as an adjuvant, and delivered via micropatch array (MPA) to the skin in comparison with more traditional routes of immunization. The micropatch contains an ultra-high density array (21,000/cm^2^) of 110 μm microprojections. Mice received 3 doses of 1 μg (nanopatch, intradermal, subcutaneous, or intra muscular injection) or 10 μg (intradermal, subcutaneous, or intra muscular injection) of tetravalent sE spaced 4 weeks apart. When adjuvanted with Quil-A, tetravalent sE vaccination delivered via MPA resulted in earlier induction of virus-neutralizing IgG antibodies for all four serotypes when compared with all of the other vaccination routes. Using the infectious dengue virus AG129 mouse infectious dengue model, these neutralizing antibodies protected all mice from lethal dengue virus type 2 D220 challenge, with protected animals showing no signs of disease or circulating virus. If these results can be translated to humans, MPA-delivered sE represents a promising approach to dengue virus vaccination.

## 1. Introduction

Since the re-emergence of dengue virus (DENV) in the 1950’s, there has been a steady increase in transmission and global disease over the intervening decades. The increasing geographic range of dengue transmission has resulted in approximately half of the world’s population living in dengue endemic regions, with an estimated 390 million dengue infections occurring annually [1]. Consequently, dengue viruses are now viewed as the most significant of all disease-causing arbovirus infections.

What makes dengue unique among flaviviruses (including Zika and West Nile virus) is that there are 4 serotypes (serotypes 1–4) that are closely related but antigenically distinct. Patients infected with any of the 4 DENV serotypes can experience a spectrum of clinical outcomes, ranging from asymptomatic to mild febrile illness through to dengue fever. Following primary infection, patients develop a life-long immunity to the original infecting virus serotype [2]. However, upon secondary infection with a heterologous serotype, in a proportion of individuals the induction of cross-reactive antibodies can lead to antibody-dependent enhancement (ADE) of infection and progression to more severe and potentially fatal disease [3].

Given the potential for re-infection with a heterotypic virus serotype leading to enhanced disease outcome, a successful dengue vaccine must be tetravalent, eliciting neutralizing antibodies for all four serotypes, such that naïve individuals are not primed for severe disease from their first encounter with wild-type virus [4]. The first, and so far only licensed DENV vaccine, Dengvaxia (Sanofi Pasteur) [5], is a mix of four chimeric, live-attenuated viruses based on the Yellow Fever virus (YFV) 17D genetic backbone, with the YFV prM and E genes replaced by the equivalent structural genes of each of the 4 DENV serotypes. While early studies proved promising, the vaccine has been shown to have significant limitations, and the WHO now recommends vaccine use be restricted only to recipients who have previously been infected with DENV [6].

Alternative strategies are being actively pursued. The DENV surface glycoprotein E is an ideal candidate for a subunit vaccine, given that it is the major target of a neutralizing antibody response [7,8,9]. However, ensuring a potent immune response remains a challenge for recombinant subunit vaccines, necessitating an effective adjuvant strategy [10]. Immune responses can be enhanced by targeting antigen presenting cells in the skin by intradermal injection or by use of microarray patches (MAPs), such as the nanopatch.

The nanopatch is a 4 × 4 mm ultra-high-density microarray patch (MAP) that contains 21,000 projections/cm^2^ of 110 μm in length. Vaccine is dry-coated onto the surface of these projections and applied to the skin using a spring-loaded applicator precisely to the epidermal and dermal layers of the skin, containing a high density of antigen presenting cells [11]. As a result of this targeted approach, our group achieved enhanced immune responses with fractional doses as compared to standard needle and syringe injection methods, such as intramuscular and subcutaneous injection. This enhancement was further boosted by the addition of an adjuvant [12]. These enhanced immune responses were observed in live-attenuated [13], DNA [14], inactivated [15,16], virus-like particle [17], conjugated [18], and split-virion vaccines [19,20].

Here, we investigate the utility of several different immunization routes with the nanopatch, namely intramuscular, subcutaneous, intradermal injection, and also intra-cutaneous delivery, for their ability to induce protective immune responses against the dengue secreted E (sE) protein.

## 2. Materials and Methods

### 2.1. Mice

Female SV129 and AG129 mice (6–8 weeks) were bred in pathogen-free conditions in the Australian Institute for Bioengineering and Nanotechnology (AIBN) animal house. All methods in this study were carried out in accordance with National Health and Medical Research Council guidelines and approved by The University of Queensland Animal Ethics committee (Approval AIBN/556/12 15/10/2014).

### 2.2. Cell Lines

Vero cells were maintained in Optimem media containing 3% fetal calf serum and penicillin/streptomycin (PenStrep).

### 2.3. DENV1-4 Viral Stocks

DENV serotypes were propagated in *Aedes albopictus* C6/36 cells before titration in Vero cells. To titer the virus for plaque reduction neutralization tests (PRNT), DENV viral stocks were serially diluted to 1:10 in optimum serum-free media and incubated for 1 h at 37 °C in an atmosphere containing 5% CO_2_. Virus was added to confluent monolayers of Vero cells in 96-well plates, seeded the previous day at a density of 4 × 10^4^ cells per well. The virus was allowed to absorb for 1 h at 37 °C in 5% CO_2_. The virus was removed before addition of 1.5% carboxymethylcellulose (CMC) overlay with M199 media (Gibco, Grand Island, NY, USA), supplemented with 2% heat-inactivated fetal bovine serum (FBS). Plates were incubated at 37 °C, 5% CO_2_ for 2 days. Cells were immunostained as described in the PRNT protocol.

### 2.4. Quantification of Mouse Viral Load by DENV Plaque Assay

Levels of free circulating virus were determined by viral plaque assay performed in Vero cells in 96-well plates. Vero cells were plated at 2 × 10^5^ cells/mL at a volume of 200 μL and allow to grow to confluency overnight at 37 °C. Cells were washed with phosphate-buffered saline (PBS) followed by serum free media. Plasma samples were serially diluted from 10^−2^ to 10^−8^ in a separate 96-well plate. After serum free media was removed from cells, 50 μL of the serially diluted virus was added to the cells and incubated at 37 °C for 2 h. Following incubation, media was removed and cells were overlaid with 200 μL of 1.5% carboxymethlycellulose (CMC) in M199 (Invitrogen) with 2.5% FCS and incubated for 4 days at 37 °C. After 4 days, the overlay was removed and cells were washed with PBS. Cells were then fixed with 200 μL of ice-cold 80% acetone/20% PBS for 20 min at −20 °C. The fixative was removed and plates were dried overnight.

Cells were then washed in PBS/0.1% bovine serum albumin (BSA) and blocked for 20 min on a shaker at room temperature in the same buffer. Following blocking, 50 μL of a 1:200 dilution of an anti-nonstructural protein 1 (NS1) rabbit polyclonal serum diluted in blocking buffer was added to each well and incubated for 1 h at room temperature. After incubation with the primary antibody, the fixed cells were washed twice in blocking buffer for 3 min on a shaker. Plates were inverted and tapped to remove wash solution between each wash step. Cells were probed with anti-rabbit Immunoglobulin G (IgG) secondary antibody (1:2000) conjugated with an 800 fluorophore. Cells were washed in phosphate-buffered saline 0.05% Tween-20 (PBS-T) 3 times and plates were allowed to dry completely before visualization and imaging on the Odyssey CLx machine (Li-Cor Biotechnology, Lincoln, CA, USA).

### 2.5. PRNT Protocol

PRNT protocol was performed as previously described with some modifications for individual viruses [21]. Vero cells were seeded in 96-well plates at a density of 4 × 10^4^ cells per well and incubated overnight at 37 °C in 5% CO_2_. Mouse sera collected during the study were heat inactivated at 56 °C for 30 min. Sera were diluted 1:25 in optimum serum-free media and serially diluted to 1:2 in a 96-well plate to a final volume of 60 μL. An equal volume of DENV virus stock diluted to a final concentration to obtain ~75 plaque forming unit (PFU)/well was added to each well to give a final serum dilution of 1:50. The serum–virus mixture was incubated at 37 °C in 5% CO_2_ for 1 h before adding 50 µL to confluent Vero cells. Plates were incubated for 1 h at 37 °C in 5% CO_2_ to allow virus absorption. Virus–serum inoculum was flicked-off and cells were washed twice in PBS before addition of 1.5% CMC overlay with M199 and 2% FBS. Plates were incubated at 37 °C, 5% CO_2_ for 2 days. This process was repeated for all four serotypes of DENV (DENV1 ET00.243, DENV2 ET00.300, DENV3 ET00.209, and DENV4 ET00.288) in duplicate for each serum sample.

CMC overlay was removed and cells were fixed in ice-cold 80% acetone/20% PBS for 15 min at −20 °C. Plates were dried completely before blocking for 30 min at room temperature in milk diluent/blocking solution (KPL) diluted in PBS 0.05% Tween 20 (PBS-T). Cells were incubated for 1 h at 37 °C with human anti-envelope monoclonal antibodies (MAbs), produced as previously described [22], and diluted in KPL blocking solution/PBS-T. For DENV serotypes 1, 2, and 3, the MAb 4E11 (1:1000) was used, while DENV4 was incubated with MAb 5H2 (1:500). Cells were washed 3 times in PBS-T and incubated with anti-human IgG secondary antibody (1:2000) conjugated with an 800 fluorophore. Cells were washed in PBS-T 3 times and plates were allowed to dry completely before visualization and imaging were performed on the Odyssey CLx machine.

### 2.6. Nanopatch Fabrication and Application

Silicon nanopatches (NP, 4 mm^2^, 21,000 projections/cm^2^ at 110 μm in length) were fabricated at the Melbourne Centre for Nanofabrication, as previously described by Jenkins et al. [23]. Vaccine and adjuvant were formulated with a 1% methycellulose and 1% trehalose solution under a nitrogen jet stream, as described by Chen et al. [24]. Coating morphology and removal were characterized by scanning electron microscopy (SEM) (Joel Neoscope, Toyko, Japan), as described by Crichton et al. [11]

### 2.7. Vaccine Delivery Efficiency

To determine the skin-delivered dose by nanopatch, we performed a ^14^C tracer assay, as described by Fernando et al. (2012) [12].

### 2.8. Immunization Study

The immunization regime was based on similar studies performed investigating candidate dengue vaccines in mouse models. Under ketamine hydrochloride (Ceva Animal Health, Glenorie, Australia) and xylazine hydrochloride (Troy Laboratories, Gendenning, Australia) sedation, SV129 mice were immunized with 1 or 0.1 μg tetravalent sE (DENV1: 258848, DENV2: PR159, DENV3: CH53489, DENV4: H241 produced from S2 cells, donated by Prof Mathew Cooper, UQ) formulation by nanopatch or 1–10 μg tetravalent sE by intradermal (ID), subcutaneous (SC), or intra muscular (IM) injection, with or without 3 μg of the saponin adjuvant Quil-A (Brenntag, Essen, Germany). Control groups received PBS delivered via each investigated injection method, while nanopatch control groups received excipients only. Nanopatches containing the dose to be delivered were applied to each ventral ear pinnae using a proprietary applicator at a velocity of 3.1 ms^−1^ and kept in place for 2 min. Mice received three doses by nanopatch or SC, ID, or IM injection at 28-day intervals, with blood samples collected one day before vaccination and 28 days after final dose.

### 2.9. DENV Challenge Study

AG129 mice received 3 doses, 4 weeks apart, of 1 µg of tetravalent sE delivered by nanopatch or ID injection, as described above. Naïve and virus control groups received PBS via ID injection while nanopatch control groups received excipients only. All groups AG129 mice were challenged 14 days after the final vaccination with 1 × 10^4^ PFU or 1 × 10^5^ PFU of mouse-adapted dengue 2 virus D220 (donated by Prof Eva Harris, UC Berkeley School of Public Health), except the naïve control, which remained uninfected as a control for mouse behavior and wellbeing. Following challenge, tail tip bleeds were collected daily for 10 days and mice were monitored twice daily for weight loss and signs of distress.

### 2.10. Anti-sE IgG Enzyme Linked Immunosorbent Assay

Subunit sE proteins of each of the 4 dengue serotypes were individually coated on to Nunc Maxisorp plates overnight at 4 °C in 50 μL of carbonate-bicarbonate buffer (pH 9.6) at 100 ng/mL. Plates were then blocked for 1 h at room temperature with 200 μL 1 x milk serum diluent (KPL, Inc., Gaithersburg, MD, USA) with 1% sucrose. Samples serially diluted in blocking buffer containing 0.05% PBS/Tween 20 (50 μL) were added to blocked plates and incubated for 1 h at 37 °C, and then washed 6 times in PBS-T. Goat anti-mouse Horseradish Peroxidase (HRP) (50 μL) diluted to 1:500 in blocking buffer with PBS-T was added to plates and incubated for 1 h at 37 °C. Following this incubation, plates were washed 6 times in PBS-T then developed using 50 μL of 3,3′,5,5′-tetramethlybenzidine (TMB) (ELISA systems) for 10 min and protected from light. The reaction was stopped by the addition of 50 μL of 1 M phosphoric acid and absorbance was read at 450 nm.

### 2.11. NS1 Quantification

The quantification of NS1 was performed as originally described by Young et al. [25], with modifications outlined by Muller et al. [26].

### 2.12. Statistical Analysis

All statistical analyses were performed using GraphPad Prism version 6.0f (San Diego, CA, USA). Multiple comparison analysis was performed using one-way analysis of variance (ANOVA), with the alpha level set at 0.05 and with a Tukey post-test.

## 3. Results

### 3.1. Nanopatch Coating and Delivery

**Figure 1:**
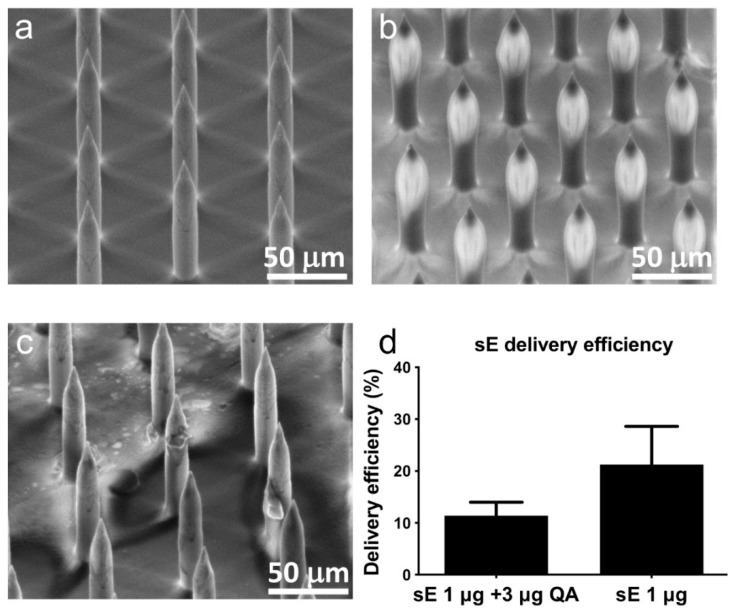
Scanning electron microscopy (SEM) analysis of (**a**) uncoated microneedles on the surface of the nanopatch: (**b**) sE coated microneedles; (**c**) the nanopatch post application, showing the removal of vaccine from the tips of the microneedle array. (**d**) As a measure of delivery efficiency, a ^14^C tracer was added to the vaccine solution to measure the bulk transfer of the nanopatch vaccine coating to the ear. The bar graph represents mean of 4–5 samples, with error bars indicating standard deviation of the mean. Note: sE = secreted E protein.

Nanopatches were coated with sE protein vaccine formulated in methylcellulose and trehalose to enhance viscosity and protect the vaccine formulation during drying, respectively. To achieve the desired delivery of 1 μg of the tetravalent sE formulation, a ^14^C tracer was added to the formulation to allow for quantitation of bulk coating deposition into the skin. Figure 1a shows the regular array of 110 μm microneedles on the surface of the nanopatch. A representative image of sE coated nanopatches is shown in Figure 1b, with the coated vaccine observed at the tips of the needles. These coated nanopatches were applied to the ear of mice (*n* = 5) to characterize sE delivery. A representative scanning electron microscope (SEM) image taken immediately after application is shown in Figure 1c, confirming the removal of the sE coating formulation from the tips of the microneedles. Transfer of the ^14^C tracer to skin was found to be 10% and 20% of the total amount loaded onto the patches for formulations, with and without Quil-A, respectively, as seen in Figure 1d, for both the formulations delivering a total of 1 μg sE (0.25 μg of each DENV serotype sE). Following the development of the coating and delivery conditions, we proceeded to test the immunogenicity of the vaccine in the SV129 mouse model.

### 3.2. sE Immunization of SV129 Mice

**Figure 2:**
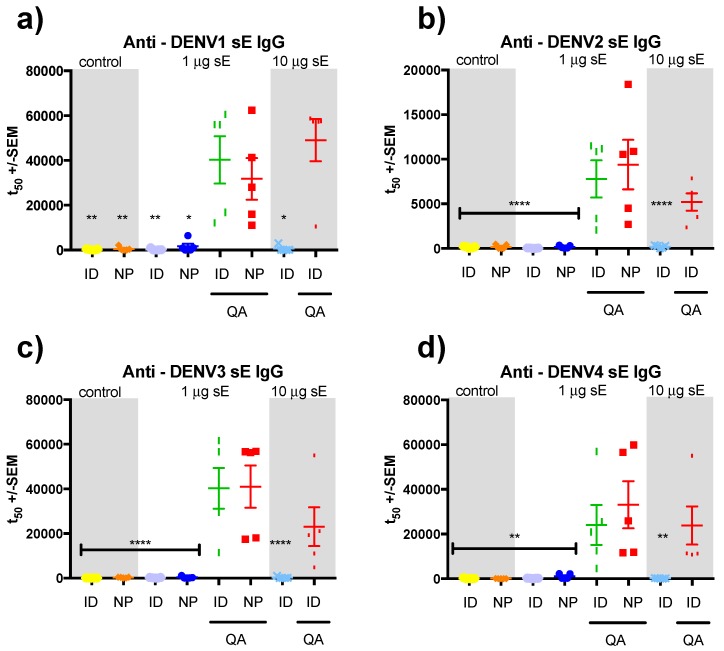
Anti-(sE) Immunoglobulin G (IgG )titers 21 days after the third immunization for SV129 mice vaccinated by nanopatch, and ID injection, with and without the adjuvant Quil-A. Samples were analyzed by anti-sE indirect Enyme-linked immunosorbent assays (ELISA) with midpoint titers shown for each serotype of DENV: (**a**) dengue 1 anti-sE responses; (**b**) dengue 2 anti-sE responses; (**c**) dengue 3 anti-sE IgG responses; and (**d**) dengue 4 anti-sE IgG responses. Each symbol represents results from a single mouse. Lines indicate mean titers, with bars indicating +/− standard error of the mean. Note: *, **, ***, and **** indicate a statistical significance for the nanopatch Quil-A group, as assessed by one-way analysis of variance (ANOVA, alpha level 0.5), of *p* < 0.5, *p* < 0.01, *p* < 0.001 and *p* < 0.0001, respectively.

Immunocompetent SV129 mice (the parental mouse line to AG129 mice) were immunized three times, 21 days apart, with one of two vaccine doses: either 1 μg (a mixed dose of 0.25 μg each serotype) or 10 μg (2.5 μg of each serotype) per 0.05 mL immunization, with and without Quil-A (3 μg). The 1 and 10 μg doses were delivered via intramuscular (IM), intradermal (ID), and subcutaneous (SC) injection with nanopatch delivery of only the 1 μg dose. The different immunization strategies were compared for induction of antigen-specific IgG responses (Figure 1 and Figure S1). Overall, similar trends were observed for the immune response to all 4 DENV serotypes (Figure 2a–d). Vaccine co-formulated to deliver 1 μg sE and 3 μg Quil-A produced significantly higher titers of DENV serotype-specific IgG when delivered by nanopatch or ID injection than by the same dose delivered via IM or SC injection (Figure S1a–d). When the dose was increased to 10 μg with 3 μg of Quil-A, the mice did not produce significantly greater IgG titers than the 1 µg dose. Indeed, significantly lower titers were seen, suggesting the possibility of high doses of sE-induced suppression of the immune response.

### 3.3. sE Nanopatch Immunization of AG129 Mice

**Figure 3:**
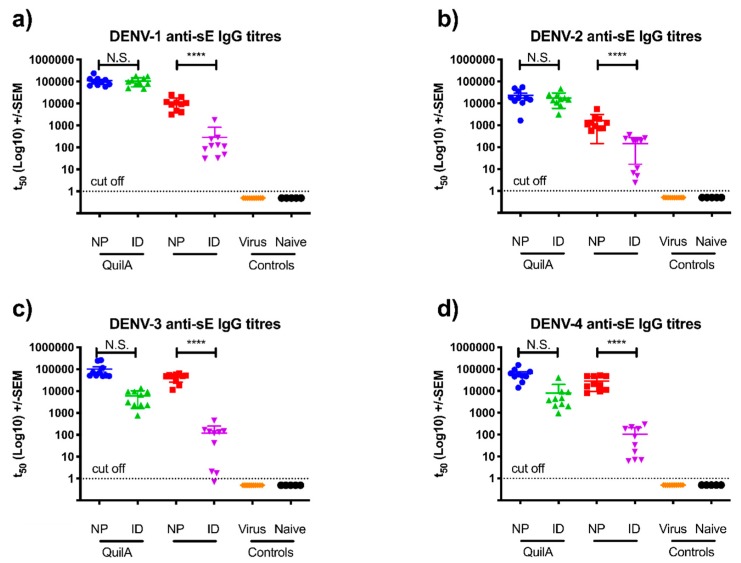
Anti-dengue sE IgG antibody titers for AG129 mice vaccinated by nanopatch and ID injection, with and without the adjuvant Quil-A, determined by indirect ELISA 14 days after the final vaccination. Here, 50% titers are shown for each serotype of DENV: (**a**) dengue 1 anti-sE responses; (**b**) dengue 2 anti-sE responses; (**c**) dengue 3 anti-sE IgG responses; and (**d**) dengue 4 anti-sE IgG responses. Each symbol represents a single mouse. Lines indicates mean titers, with bars indicating +/− standard error of the mean. Note: *, **, ***, and **** indicate a statistical significance for the nanopatch Quil-A group, as assessed by one-way analysis of variance (ANOVA, alpha level 0.5), of *p* < 0.05, *p* < 0.01, *p* < 0.001, and *p* < 0.0001, respectively.

In order to examine the protective efficacy of the immune responses elicited, we utilized the established AG129 (interferon α/β, and γ receptor knock-outs) mouse challenge model [27,28]. The immunogenicity analysis was repeated in AG129 mice (n = 10) using all four DENV sE serotypes, and focusing on the comparison between nanopatch and ID delivery routes, given their clear superiority over IM and SC routes (Figure S1), as we have reported previously [29]. We observed (Figure 3a–d) similar levels of antibody responses across all four serotypes, regardless of the delivery route, confirming that the tetravalent antigen induced comparable IgG titers independent of virus serotype. With the change of mouse model, we observed increased antibody titers for those mice immunized without adjuvant, regardless of the delivery route. In the absence of adjuvant, the nanopatch yielded higher antibody titers in comparison to ID, however in the presence of Quil-A, antibody titers were consistent for both nanopatch and ID routes following the final dose (Figures S2–S4).

**Figure 4:**
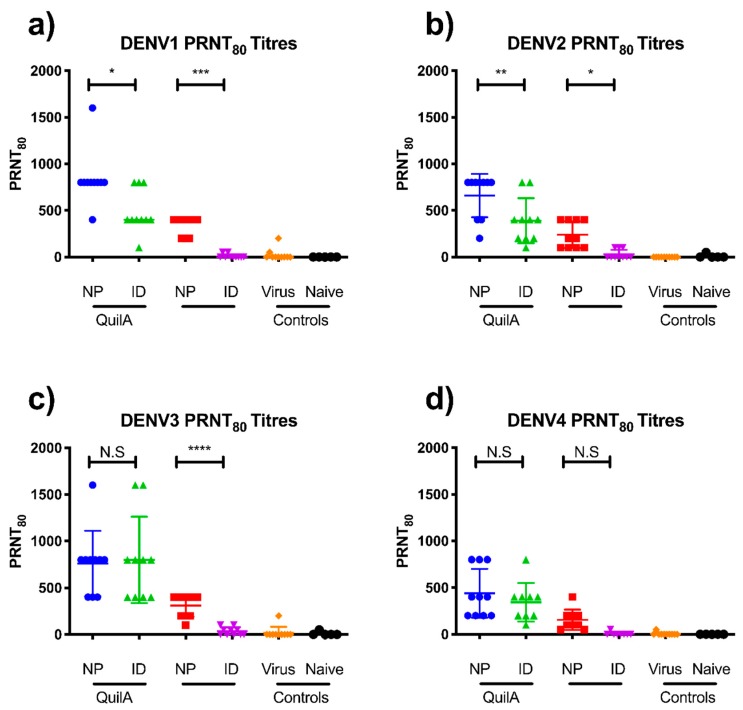
Virus-neutralizing antibody titers for AG129 mice vaccinated by nanopatch and ID injection, with and without the adjuvant Quil-A 14 days after the third vaccination. PRNT_80_ titers are shown for each DENV serotype: (**a**) DENV1; (**b**) DENV2; (**c**) DENV3; and (**d**) DENV4. Each symbol represents a single mouse. Lines indicate mean titers, with bars indicating +/− standard error of the mean. Note: *, **, ***, and **** indicate a statistical significance for the nanopatch Quil-A group, as assessed by one-way ANOVA (alpha level 0.5), of *p* < 0.05, *p* < 0.01, *p* < 0.001, and *p* < 0.0001, respectively.

In addition to the anti-sE IgG analysis, we performed plaque reduction neutralization (PRNT) assays to determine the presence of virus-neutralizing antibodies. As seen in Figure 4a–d, the PRNT_80_ titers showed consistent trends with the anti-sE IgG analysis. Again, vaccination with the tetravalent sE formulation produced a balanced response, with similar mean neutralizing titers observed for all serotypes. Unlike the IgG analysis, a significant difference was observed between matched ID and nanopatch groups, suggesting a greater proportion of virus-neutralizing antibodies in the antibody response of animals receiving the nanopatch-delivered vaccine.

### 3.4. DENV Challenge of Vaccinated AG129 Mice

**Figure 5:**
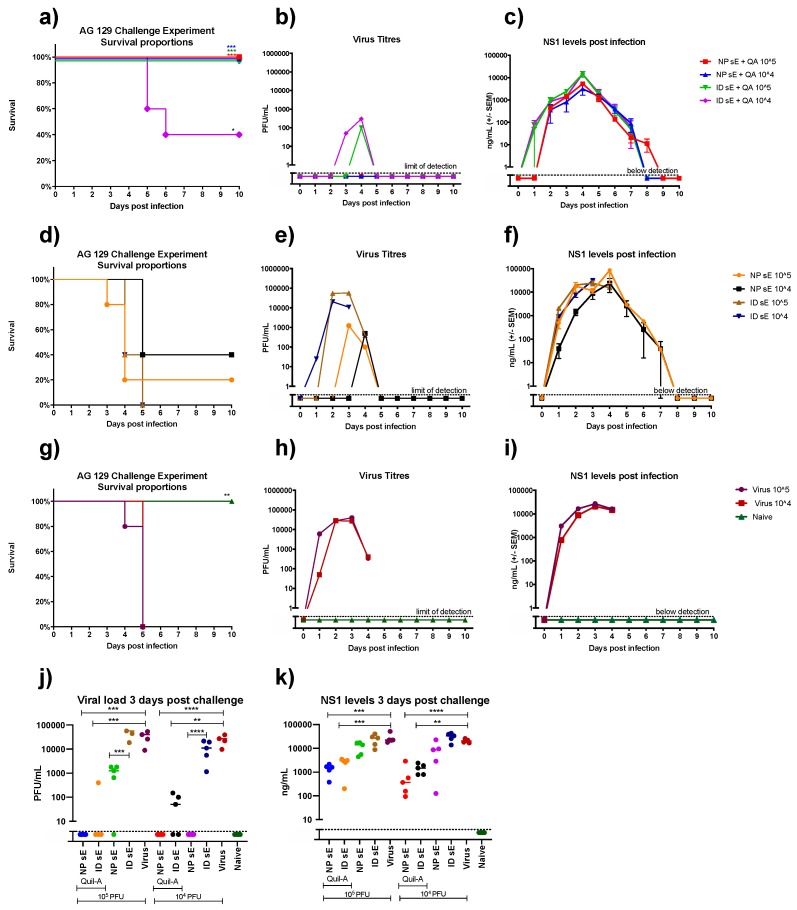
Survival rates, viral titers, and NS1 concentration in mice infected with 1 × 10^5^ or 1 × 10^4^ PFU of DENV2 D220 10 days after the final tetravalent sE vaccination. Mice were monitored daily for survival rate (**a**,**d**,**g**), with blood samples collected daily from days 1–10 for analysis. The difference between vaccinated groups and virus control groups was determined by log-rank (Mantel-cox test). Daily samples were analyzed for the presence of circulating virus by plaque assay in Vero cells (**b**,**e**,**h**). Plasma levels of NS1 were also determined by quantitative capture ELISA (**c**,**f**,**i**). To demonstrate the significant reduction in virus titer (**j**) and NS1 titers (**k**) resulting from vaccination, the raw data for day 3 post-virus challenge is presented. Note: *, **, *** and **** indicate a statistically significant reduction from the in virus titer and NS1 titers, as assessed by one-way ANOVA (alpha level 0.5). of *p* < 0.05, *p* < 0.01, *p* < 0.001, and *p* < 0.0001, respectively.

Given the strong neutralizing antibody responses induced by nanopatch and ID vaccination in AG129 mice, we next evaluated the degree of protection elicited by a lethal virus challenge. Groups of 10 mice were randomly split into two groups of five for challenge with either 10^4^ or 10^5^ plaque-forming units (PFU) of the mouse-adapted D220 DENV2 strain (equivalent to one or 10 times the experimentally-determined LD50). Aside from monitoring symptoms of infection by scoring weight loss (Figure S5) and changes in mouse behavior, we measured viral titer and DENV NS1 levels in plasma to determine the effect of vaccination on viral replication. For unvaccinated control mice, challenge with 10^4^ or 10^5^ PFU resulted in a rapid increase in viral load and NS1 levels over the first 3 days, with mice in a moribund state humanly culled by days 4–5 (Figure 5g–i). Mice vaccinated with sE without adjuvant showed mixed results based on the vaccine delivery route. All mice vaccinated via the ID route succumbed to virus challenge, and had higher circulating viral titers in comparison to the nanopatch-immunized group. In contrast, the nanopatch-immunized group had partial protection from both doses of lethal virus challenge, with significantly lower circulating viral titers (Figure 5d–f,i). In the case of adjuvanted vaccinations, 100% of animals survived following nanopatch plus Quil-A immunization, with no virus breakthrough observed (Figure 5i) and significantly reduced NS1 levels (Figure 5j) as compared to the controls (Figure 5a–c). Partial protection was afforded to mice immunized with sE plus adjuvant via the ID route, suggesting that the presence of the adjuvant significantly improved both the immune responses and the degree of protection from lethal virus challenge (Figure 5a). Both viral and NS1 titers were reduced in these groups, consistent with the improved immune response and survival outcome (Figure 5a–c).

## 4. Discussion

Here, we describe a potent, tetravalent dengue subunit vaccine that induces enhanced levels of neutralizing antibody to all 4 DENV serotypes when delivered by nanopatch compared to other traditional immunization routes. Mice immunized by nanopatch with 1 μg of tetravalent sE formulated with the adjuvant Quil-A were afforded complete protection in a mouse challenge model. If this approach in mice translates to similar efficacy in humans, this could result in a highly protective vaccine coupled with the practical advantages conferred by the nanopatch delivery platform.

The licensing of the Sanofi dengue chimeric vaccine was an important step in dengue vaccine development. However, given the age restrictions and specific conditions under which the vaccine has been recommended for use, there remains an urgent need for further DENV vaccine development. An array of approaches are currently underway, with additional chimeric vaccines in advanced development by Takeda and National Institutes of Health. Subunit vaccine approaches based on the main target of a neutralizing antibody response, the DENV E glycoprotein, also have great potential [7]. Despite previous evidence that the DENV E protein is poorly immunogenic on its own [7,10], there are several practical advantages in the subunit vaccine approach over live-attenuated vaccines. These include safety, flexibility to adjust the antigenic dose to balance the immune response, and the ability to safely immunize infants and immunosuppressed individuals [30].

To overcome the weakly immunogenic nature of the E protein, we included Quil-A adjuvant, which we have previously shown significantly enhances immune responses in small animal models [12,18,31,32]. The formulated vaccines were then dry-coated on the nanopatch for delivery. The nanopatch provides several advantages over traditional needle and syringe delivery, particularly in developing world and resource-limited settings. These include the absence of a need for specialized medical personnel to administer the vaccine, no sharp waste being generated, and no need for vaccine reconstitution [33].

In SV129 mice, a balanced IgG response was observed for DENV1, DENV3, and DENV4, however IgG titers were significantly lower in mice immunized with DENV2 sE. This finding is in contrast with a previous study carried out in BALB/c mice using intramuscular immunization of sE, which reported a more balanced IgG response with a vaccine formulation comprising a molar ratio of 1:1:1:2 of DENV1/2/3/4 sE, respectively [34,35]. The serotype bias differences between these studies may be due to mouse strain variation, given that the switch to the AG129 mouse model led to a more balanced IgG immune response following immunization with an equimolar amount of DENV1-4 sE. Immunization with 1 μg tetravalent sE with and without Quil-A elicited similar antibody titers against all dengue serotypes using either NP or ID routes. As seen previously, Quil-A significantly increased both IgG and neutralization titers (Figure 3 and Figure 4). Quil-A is a highly effective adjuvant in small animal models and is used here to assess the role of adjuvants in eliciting an effective protective immune response. However, Quil-A is not suitable for vaccine use in humans, and alternative adjuvants will be screened in future studies. These include QS-21, a purified derivative of Quil-A that is licensed for use in conjunction with monophosphoryl lipid A (AS01b) as an adjuvant to the shingles vaccine, Shingerix [36]. To translate our findings beyond these preliminary preclinical evaluation studies, careful consideration of adjuvant choice will be required. While beyond the scope of this study, DENV serotype antigen ratios and epitope bias will need to be evaluated to ensure a balanced, elicited antibody response that takes into consideration the important issue of ADE.

While there has been intensive research over many decades characterizing the anti-dengue immune response to various candidate dengue vaccines, there have only been a limited number of studies performed using interferon receptor knockout AG129 (interferon α/β, γ receptor knockout) or interferon α/β receptor knockout (INFAR1) mice. The attraction of these interferon receptor knockout mice are that they are receptive to DENV infection via peripheral challenge with resulting viremia and relevant pathology. However, their IFN-deficient status presents some caveats to the interpretation of immune responses. Nevertheless, immune responses elicited in these animal models to various vaccine formulations have previously been shown to induce some level of protection from DENV challenge [37,38]. To the best of our knowledge, the current study is the first to assess the potential of sE subunit formulations to afford protection from DENV challenge in the AG129 mouse model. Mice immunized by NP delivery with sE and formulated with Quil-A showed no signs of virus infection, with all mice surviving. Furthermore, upon analysis of plasma samples collected over the course of infection, no viremia breakthrough was observed. It is notable that NS1 was still detected as circulating in the blood of these protected animals, albeit at slightly reduced levels, and with peak levels delayed by a day when compared to control infected mice. This suggests ongoing virus replication and perhaps further immune stimulation in the absence of detectable virus circulation.

This is the first study to demonstrate effective microarray patch delivery of a tetravalent DENV sE vaccine. Immune responses to all DENV serotypes were elicited and protection against DENV-2 challenge was demonstrated in the AG129 mouse model. The highly effective nature of the nanopatch array delivery system warrants further evaluation in larger animal models and with consideration for other flaviviruses, including yellow fever and emerging threats such as Zika virus.

## Supplementary Materials

The following are available online at https://www.mdpi.com/2076-393X/7/4/189/s1: Figure S1: Complete data set of anti-sE IgG titers from SV129 mice vaccinated by nanopatch, ID, SC, and IM injection, with and without the adjuvant Quli A. Figure S2: AG129 anti-sE IgG ELISA results post dose 1. Figure S3: AG129 anti-sE IgG ELISA results post dose 2. Figure S4: AG129 anti-sE IgG ELISA results post dose 3. Figure S4: Percentage weight loss from AG129 mice following DENV D220 challenge.
