# Supplementary material for: Efficient Delivery of Dengue Virus Subunit Vaccines to the Skin by Microprojection Arrays
Source: Vaccines (Basel). 2019 Nov 20;7(4):189. doi: 10.3390/vaccines7040189 (PMC6963636; doi:10.3390/vaccines7040189)
Supplement: Supplementary file 1 [file vaccines-07-00189-s001.pdf]

Supplementary figures

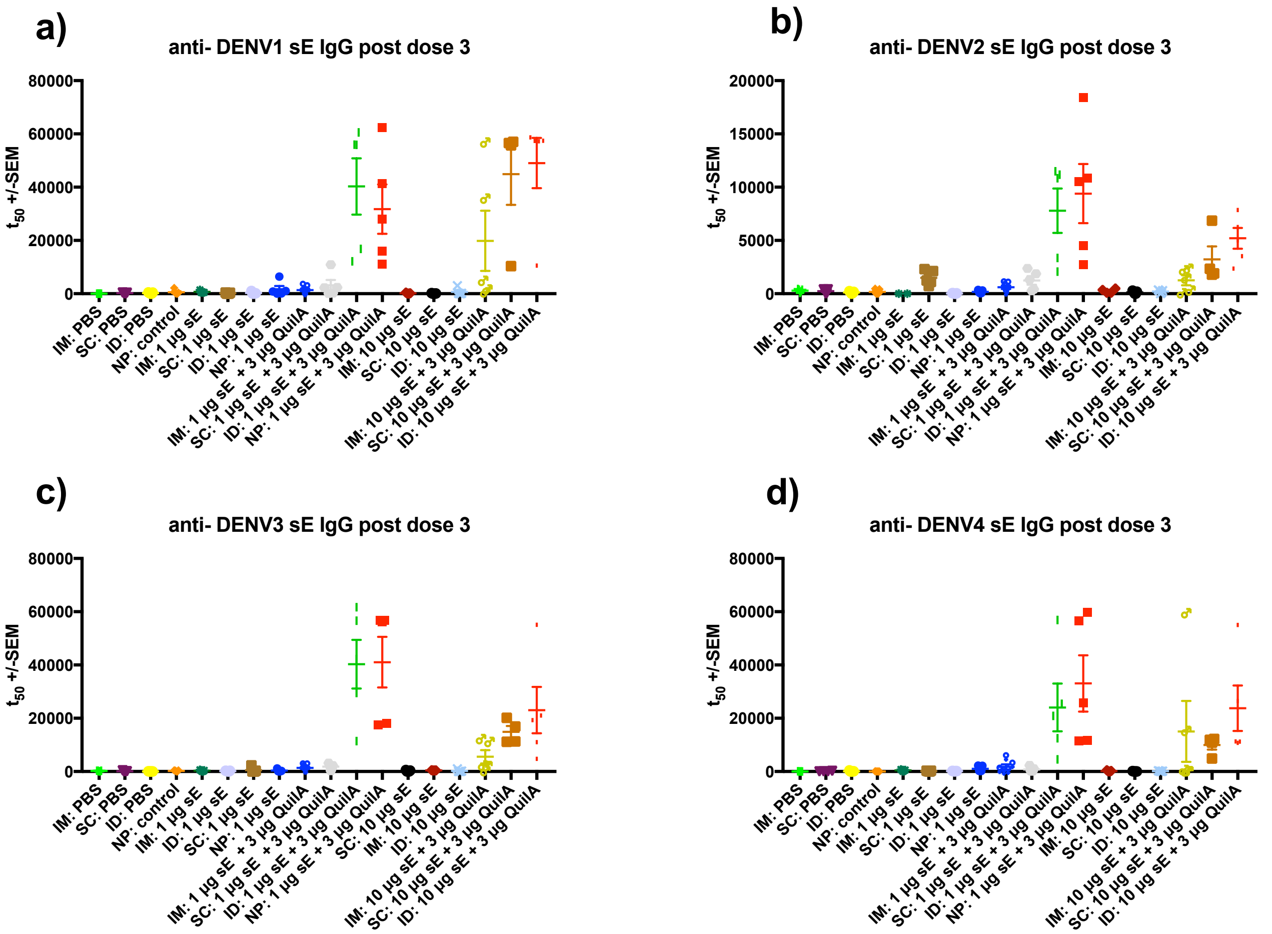


**Figure s1:** Complete data set of anti-sE IgG titres for SV129 mice vaccinated by Nanopatch, ID, SC and IM injection with and without the adjuvant Quil-A. 50% titres calculated from sera samples collected 4 weeks following the final dose. Sera was analyzed against sE from each serotype of dengue virus. **(a)** dengue 1 anti-sE responses **(b)** dengue 2 anti-sE responses **(c)** dengue 3 anti-sE IgG responses and **(d)** dengue 4 anti-sE IgG responses. Each symbol represents a single mouse. Lines indicate mean titres with bars indicating +/- standard error of the mean.

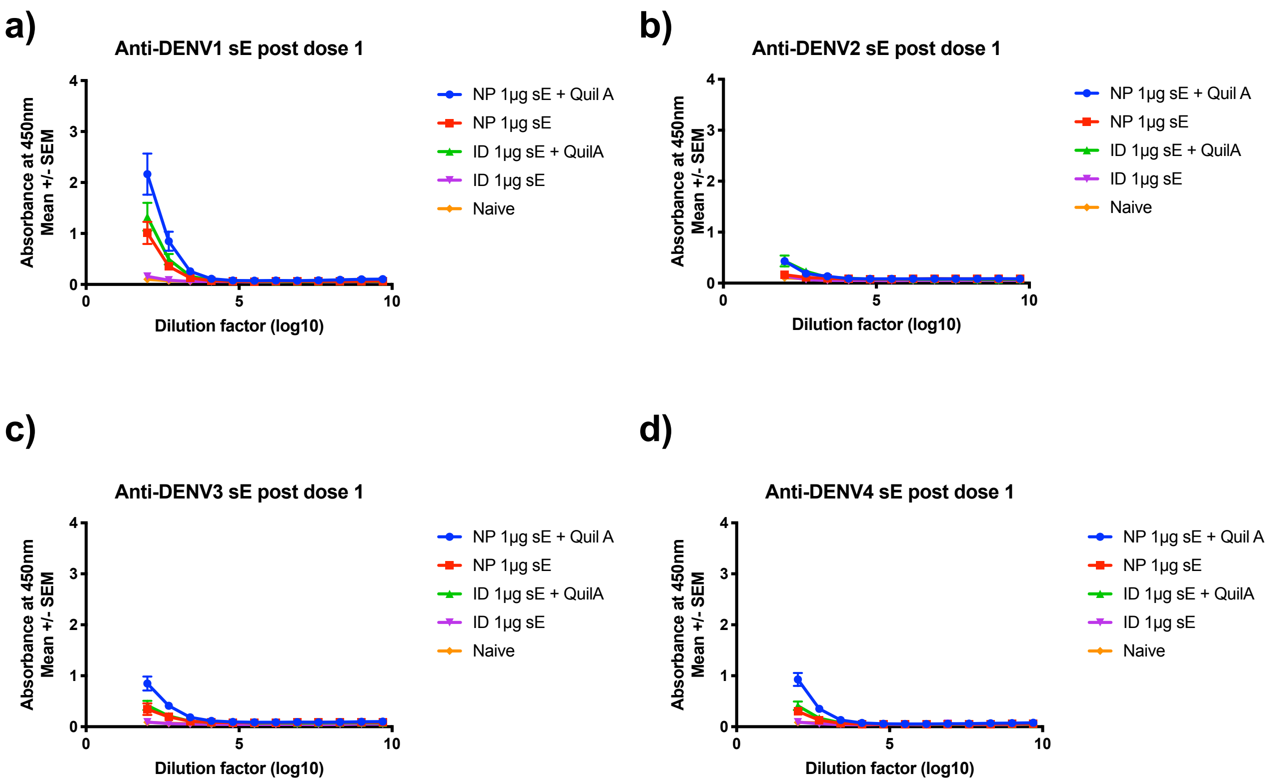


**Figure s2:** AG129 anti-sE IgG ELISA results post dose 1 **(a)** dengue 1 anti-sE responses **(b)** dengue 2 anti-sE responses **(c)** dengue 3 anti-sE IgG responses and **(d)** dengue 4 anti-sE IgG responses. Each line represents the mean absorbance of all mice (n=10) within a group. Bars indicating +/- standard error of the mean.

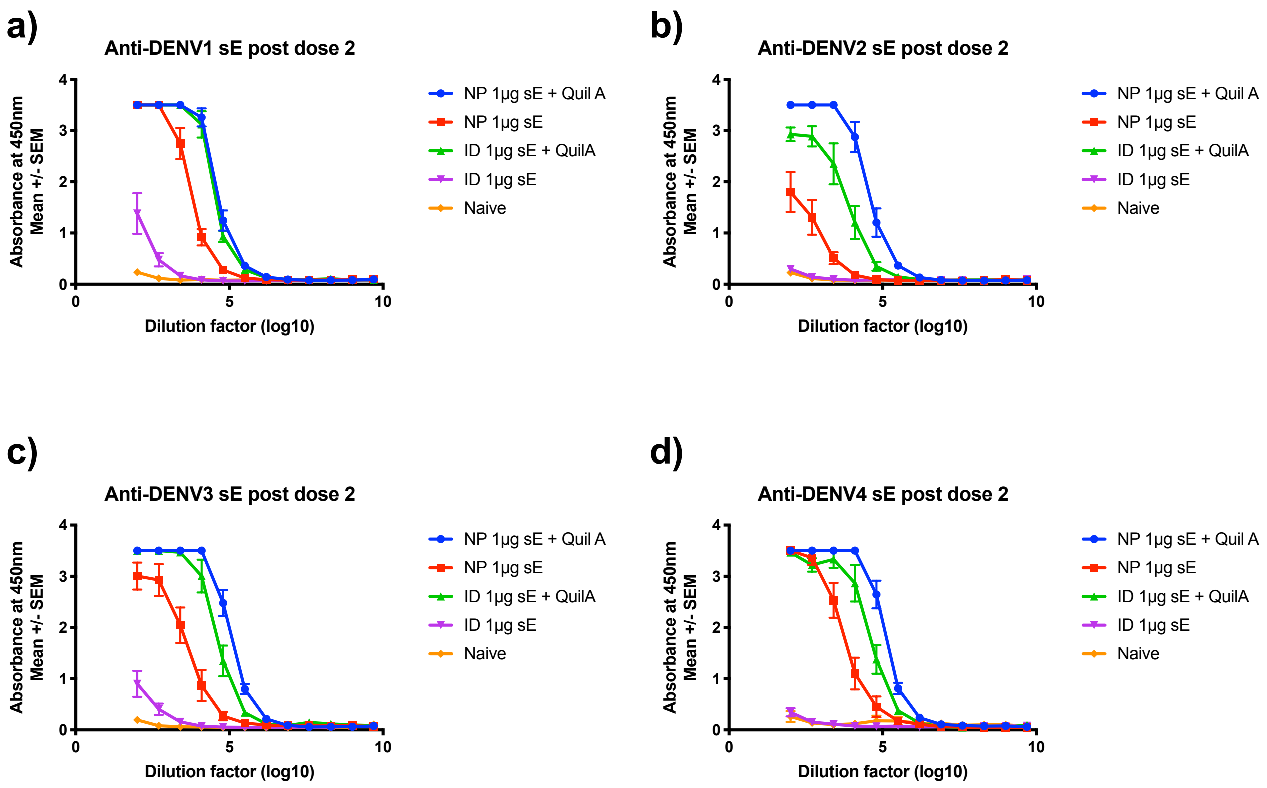


**Figure s3** AG129 anti-sE IgG ELISA results post dose 2 **(a)** dengue 1 anti-sE responses **(b)** dengue 2 anti-sE responses **(c)** dengue 3 anti-sE IgG responses and **(d)** dengue 4 anti-sE IgG responses. Each line represents the mean absorbance of all mice (n=10) within a group. Bars indicating +/- standard error of the mean.

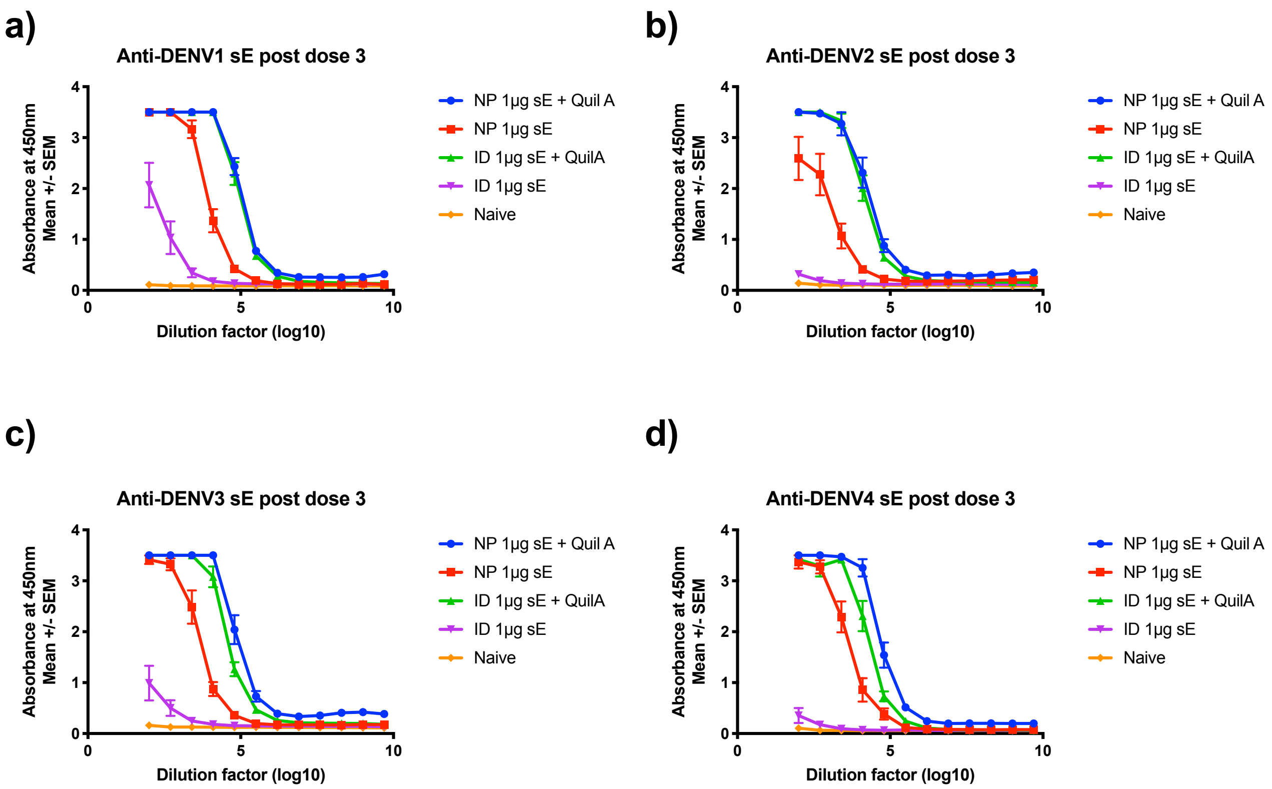


**Figure s4** AG129 anti-sE IgG ELISA results post dose 3 **(a)** dengue 1 anti-sE responses **(b)** dengue 2 anti-sE responses **(c)** dengue 3 anti-sE IgG responses and **(d)** dengue 4 anti-sE IgG responses. Each line represents the mean absorbance of all mice (n=10) within a group. Bars indicating +/- standard error of the mean.

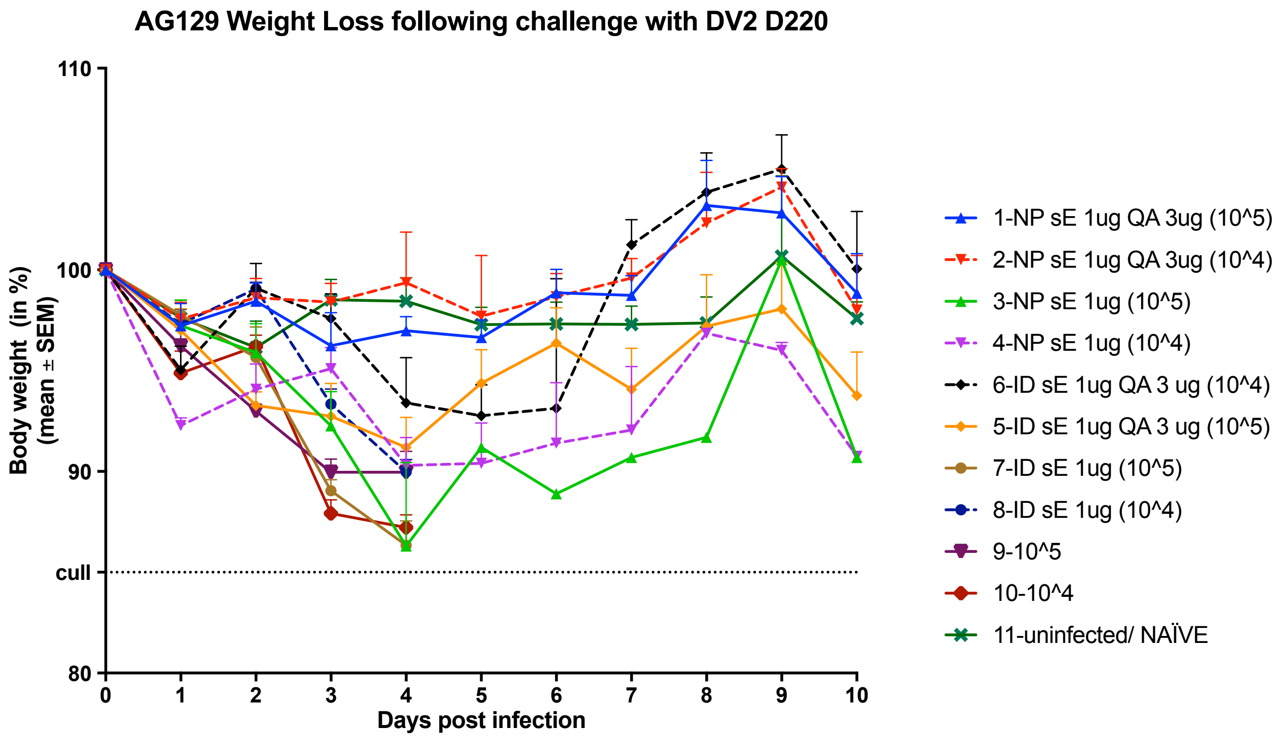


**Figure s5:** Percentage weight loss from AG129 mice following virus DENV220 challenge. Plotted is the mean percentage weight loss per group (n=5) Dotted line represents 15% weight loss. Bars indicating +/- standard error of the mean.
